# 2D-Qsar for 450 types of amino acid induction peptides with a novel substructure pair descriptor having wider scope

**DOI:** 10.1186/1758-2946-3-50

**Published:** 2011-11-02

**Authors:** Tsutomu Osoda, Satoru Miyano

**Affiliations:** 1 Department of Information Science and Technology, The University of Tokyo, Shiroganedai 4-6-1, Minato-ku, Tokyo, Japan

## Abstract

**Background:**

Quantitative structure-activity relationships (QSAR) analysis of peptides is helpful for designing various types of drugs such as kinase inhibitor or antigen. Capturing various properties of peptides is essential for analyzing two-dimensional QSAR. A descriptor of peptides is an important element for capturing properties. The atom pair holographic (APH) code is designed for the description of peptides and it represents peptides as the combination of thirty-six types of key atoms and their intermediate binding between two key atoms.

**Results:**

The substructure pair descriptor (SPAD) represents peptides as the combination of forty-nine types of key substructures and the sequence of amino acid residues between two substructures. The size of the key substructures is larger and the length of the sequence is longer than traditional descriptors. Similarity searches on C5a inhibitor data set and kinase inhibitor data set showed that order of inhibitors become three times higher by representing peptides with SPAD, respectively. Comparing scope of each descriptor shows that SPAD captures different properties from APH.

**Conclusion:**

QSAR/QSPR for peptides is helpful for designing various types of drugs such as kinase inhibitor and antigen. SPAD is a novel and powerful descriptor for various types of peptides. Accuracy of QSAR/QSPR becomes higher by describing peptides with SPAD.

## Background

Research on the classification of small molecules using computers was popular in the 1990s [1-5], with similarity analysis of compounds being a major objective. At the time, there were mainly two methods for similarity analysis: the fingerprint description approach [4,6] and the inductive logic programming approach [7-9]. In the fingerprint description approach, a molecule is described as a sequence of bits, each of which corresponds to the existence of a chemical substructure. Atom-pair descriptor [4] or substructure type fingerprints are popular descriptors.

Research on the classification of peptides became popular in the year 2000 [10-12]. The hidden Markov model (HMM) approach [12] and physical data description of peptide approach [11] were the major approaches. The main subject of these papers is the natural twenty amino acids, such as isoleucine, valine, and so on. For example, the subject of immunity concerns peptides whose components are one of 20 natural amino acids. In traditional research for the classification of peptides, an amino acid residue was described as an alphabet or a set of physical or chemical values [11].

However, in practical virtual screening, describing other amino acid inductions such as cyclohexyl alanine or F5 phenylalanine is necessary. The traditional description of peptides is not sufficiently powerful because the common characteristics among amino acid residues cannot be described sufficiently. For example, tyrosine and phenylalanine have an aromatic ring substructure in common. In the alphabetic description, tyrosine and phenylalanine are described as 'Y' and 'F' respectively. However, understanding that symbols 'Y' and 'F' have a common substructure on a machine learning algorithm is impossible. Research of two-dimensional QSAR has been undertaken for various types of peptides. In the atom-pair holographic code (APH) [13], each peptide is described with the method similar to atom-pair descriptor [3]. Our novel descriptor, substructure-pair descriptor (SPAD), captures different characteristics of peptides from APH and has greater descriptive power than APH. The combination of APH and SPAD may lead to better QSAR for peptides with many types of amino acid inductions [14].

Tanimoto coefficient [15] is a popular indicator for measuring similarity between two compounds [16]. In binary case, Tanimoto coefficient *T*(*X*, *Y *) between vectors *X *and *Y *is defined as following expression.

$$\begin{array}{lll} X & =\left(x_{1},x_{2},\cdot \cdot \cdot ,x_{n}\right),\;x_{k}=0\;or\;1,\;1\leq k\leq n\; & \text{(1)}\\ Y & =\left(y_{1},y_{2},\cdot \cdot \cdot ,y_{n}\right),\;y_{k}=0\;or\;1,\;1\leq k\leq n\; & \text{(2)}\\ T\left(X,Y\right) & =\frac{\sum_{k=1}^{n}x_{k}y_{k}}{\sum_{k=1}^{n}max\left(x_{k},y_{k}\right)}\; & \text{(3)}\\ & \; & \text{(4)} \end{array}$$

Tanimoto coefficient becomes large when two vectors have more similar bit-pattern. When the structure of two compounds is similar, Tanimoto coefficient is also high.

In machine learning, excessive features degrade the performance of machine learning algorithms due to over-fitting problems [17]. Under excessive feature space, predictive models lose robustness. Feature selection is necessary for building more accurate predictive models. Kohavia proposed the relevance of features instead of maximizing accuracy of an algorithm [18]. Discussions about relevance of features are popular in various types of algorithm [19]. Relevance is defined as the difference between probability density function *P*(*Y *= *y*) and conditional probability density function *P*(*Y *= *y*|*X_i _*= *x_i_*). When *P*(*Y *= *y|X_i _*= *x_i_*) ≠ *P*(*Y *= *y*), *X_i _*is relevant. Otherwise, *X_i _*is irrelevant.

In information theory [20], entropy is an indicator for measuring the amount of information. We denote probability of *x_i _*as *P*(*x_i_*). Entropy *E *is defined as next function.

$$E=\overset{n}{\underset{i=1}{\sum }}P\left(x_{i}\right)\log P\left(x_{i}\right)$$

## Methods

### Definition of several terms

In this paper, we define several terms as follows.

• Substructure: a part of structure of peptides

• Descriptor: The function for mapping a structure of amino acid residues or peptides to a bit according to substructure.

• Feature: A bit as the result of a descriptor.

A target protein binds some amino acid residues of peptides by some kinds of chemical or physical interactions. For example, hydrogen bonds and hydrophobic effect are representative interactions. In our QSAR approach, we describe the two-dimensional structure of peptides with a sequence of bits and analyze the relationship between peptides structure and its activity statistically. When we analyze this relationship with a data mining algorithm, QSAR rules are extracted automatically from dataset annotated with peptides' activity. From a chemical viewpoint, describing various types of amino acid inductions properly is important for improving QSAR analysis.

From a statistical viewpoint, features which maximize the accuracy of an algorithm for analyzing QSAR are the best. Kohavi proposed the relevance of features instead of maximizing accuracy of an algorithm. Discussions about relevance of features are popular in various types of algorithm [19]. Relevance is defined as the difference between probability density function *P*(*Y *= *y*) and conditional probability density function *P*(*Y *= *y*|*X_i _*= *x_i_*). When *P*(*Y *= *y*|*X_i _*= *x_i_*) ≠ *P*(*Y *= *y*), *X_i _*is relevant. Otherwise, *X_i _*is irrelevant.

**Figure 1:**
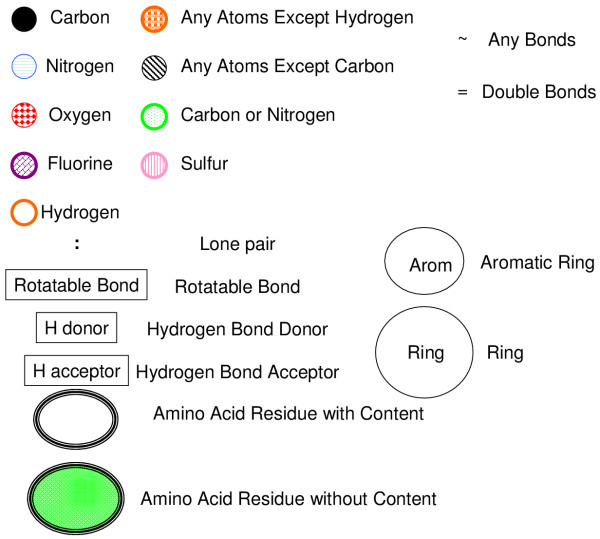
**Definition of symbols**.

We define each symbol as Figure 1. The SPAD is defined with these symbols.

### Definition of the base substructure set for amino acid inductions

**Figure 2:**
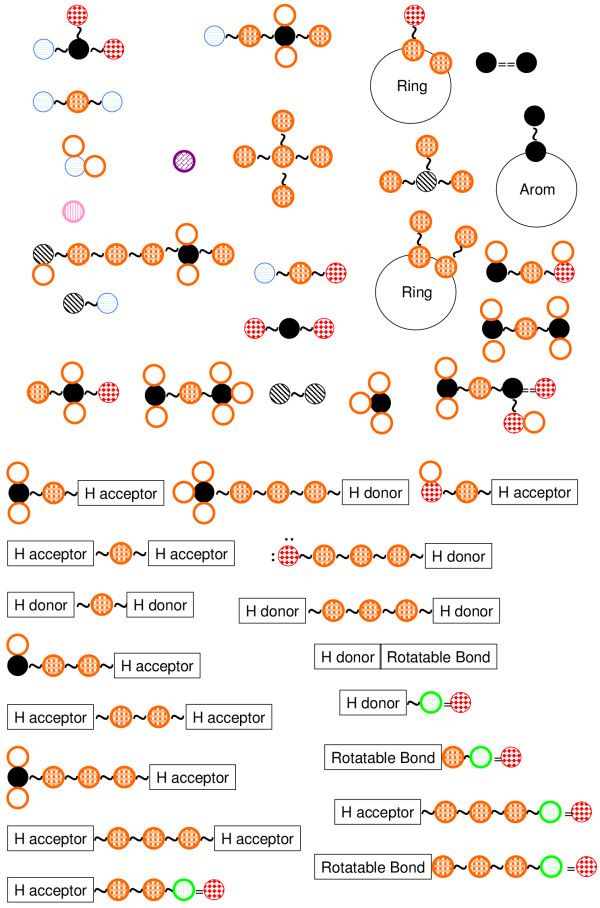
**Definition of a set of base substructures in SPAD, which roughly has three categories, i**.e., number of atoms, substructures (above), and peptide properties (below). Number of atoms includes 'Cl', 'F', 'N', 'O', 'C', 'C in aromatic ring', 'S', 'N in aromatic ring' and 'Sum of left atoms'.

The aim of defining the base substructure (Figure 2) set is the description of important interactions between a target protein and a peptide such as hydrogen bonds, the hydrophobic effect, and so on. However statistically redundant or specific descriptor may degrade the accuracy of an algorithm for QSAR analysis. We defined the base substructure set under next three conditions.

• Describe potential factors for interactions such as hydrogen bond acceptor.

• Features of amino acid residues should be weak relevant to each other mathematically. This is the condition for avoiding strong relevant features. Abandon features with strong relevance.

• A feature should have high entropy (in information theory) after mapping structures of 450 types amino acids to a sequence of bits. This is the condition for avoiding too specific descriptor. Abandon descriptors with low entropy.

The first item is essential for QSAR analysis because key substructures such as hydrogen bond acceptor may cause the activity of peptide for target protein. Under the condition lack of description of them, most of algorithms analyzing QSAR become powerless. The second and third items are necessary for efficient analysis from a statistical viewpoint. The second item prohibits the redundancy of features. Even if the structures of two amino acid inductions are chemically different, two features may be relevant to each other. Then, these two features are redundant statistically. The third item is necessary for generating robust QSAR rules. Features with low entropy (in information theory) lose generality.

The set of substructures *Z *includes the forty-nine substructures shown in Figure 2. These substructures are roughly categorized into three parts. Three categories are "the number of atoms", "Substructures" and "Properties". The number of atoms indicates how many atoms there are in an amino acid residue. "Substructures" indicates whether an amino acid residue has a specific substructure or not. "Properties" indicates whether an amino acid residue has some character from a viewpoint. For example, the first item of "Properties" describes the structure that is the methylene group and a hydrogen bond acceptor are connected via any atom.

An element *z *∈ *Z *denotes each substructure shown in Figure 2. Then, we can define any substructures except *z *as z*. In other word, each element z* is defined corresponding to each *z*. The substructure z* is complement of the substructure of *z *because *z *∩ z* = *ϕ*, *z *∪ z* = *All*. Then, we define the set Z* as all elements z*. Finally, we define the base substructure set *X *as *X *= *Z *∪ Z*.

### Definition of a set of intermediate bindings between any two base substructures

The activity of a peptide is determined not only by the structure of each amino acid residue but also by the relationship among amino acid residues. Here, we define an intermediate binding between two amino acid inductions as the distance between any two base substructures.

The definition of intermediate bindings among base substructures is arbitrary. For example, we can define an intermediate binding among three base substructures. When we describe the relationship among *m *substructures, the number of combinations is *O*(*n^m^*). Here, *n *is the number of substructures. The number of combinations increases by exponential order. To avoid the exponential order, we limited the number of substructures to 2.

Structures of peptides are more flexible than small compounds because peptides have many rotatable bonds. Descriptors for peptides should have a potential for describing the flexibility to obtain high accuracy.

**Figure 3:**
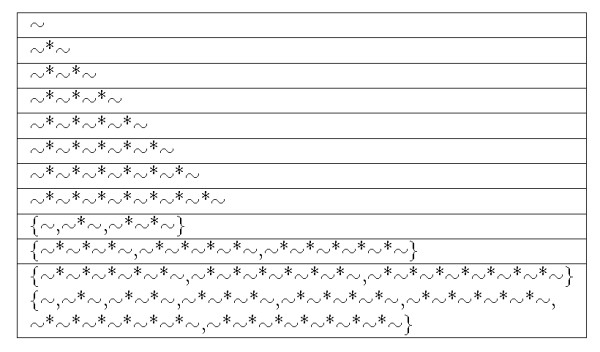
**Definition of a set of intermediate bindings in SPAD**. Intermediate bindings between two substructures are shown.

We defined the intermediate bindings shown in Figure 3. To increase flexibility of descriptors, we added a set of bindings within some length to the definition. In Figure 3, '*' denotes an amino acid residue and '~' denotes a peptide binding. '{}' denotes 'or ' condition. For example, '{~, ~ *~, ~*~*~}' represents the peptide consisting of amino acid residues from 0 to 2. We represent a set of intermediate bindings as set *Y*.

### Definition of substructure-pair descriptor

Then, SPAD is defined as next function. We suppose that the number of *X *is *N *and that the number of *Y *is *M*.

$$\begin{array}{lll} x_{i},\;x_{j}\in X,\;y_{k}\in Y & , & 1\leq i,j\leq N,\;1\leq k\leq M\\ F\text{(}x_{i}\text{,}\;\;y_{k}\text{,}\;x_{j},\;p_{a}) & = & \begin{array}{l} 1\;if\;apptide\;p_{a}\;has\;the\;structure\;\\ where\;substructures\;x_{i}\;and\;x_{j}\;are\;connected\;each\;\\ other\;via\;the\;intermediate\;binding\;y_{k}.\;In\;this\;case,\\ x_{i}\;and\;y_{k}\;or\;x_{j}\;and\;y_{k}\;are\;not\;always\;neighboring.\\ However,\;the\;number\;of\;amino\;acid\;residues\\ between\;two\;substructures\;x_{i}\;and\;x_{j}\;must\;be\;equal\\ to\;the\;length\;of\;y_{k}. \end{array}\\ F\text{(}x_{i}\text{,}\;y_{k}\text{,}\;x_{j},\;p_{a}) & = & 0\;Otherwise \end{array}$$

When *x_i_*, *x_j _*and *y_k _*are given, a peptide *p_a _*is converted to a bit with function *F *(*x_i_*, *y_k_*, *x_j_*, *p_a_*). Here, we denotes the suffix set (i, j, k) as *b*. Then, we obtained the matrix (*M_ab_*) = (*F *(*x_i_*, *y_k_*, *x_j_*, *p_a_*)) for the input of QSAR analysis algorithm. The vector (*M*_*a*1_, *M*_*a*2_, ⋯) is corresponding to the features of the peptide *p_a_*.

## Results and Discussion

### Definition of Datasets

We use two types of datasets for evaluation of the proposed descriptors. One is C5a inhibitors [21] and the other is kinase inhibitors [22]. Positive data are defined as peptides with high inhibitory potential, and negative data are defined as other peptides and peptides with random arrays. Content of dataset is as follows.

• C5a Inhibitors:

- The number of positive peptides: 116

- The number of negative peptides: 451

• kinase inhibitors:

- The number of positive peptides: 24

- The number of negative peptides: 325

### Difference between SPAD and APH definition

SPAD is different from APH in defining whether any two substructures are connected directly to an intermediate binding. For example, when the main chain is connected to an aromatic ring of a side chain via a carbon chain and two amino acid residues have carbon chains which are different to each other in its length, APH classifies two amino acid residues. However, SPAD does not. The structures of amino acid residues are very similar so it is natural to consider that their properties are approximately similar. In this case, the descriptor that ignores the difference is better. The second different point between SPAD and APH is whether the information about properties is included in descriptors. It may be unnecessary to distinguish amino acid residues from a viewpoint of some property.

#### Comparison of descriptors correlated highly with peptides' activity

**Figure 4:**
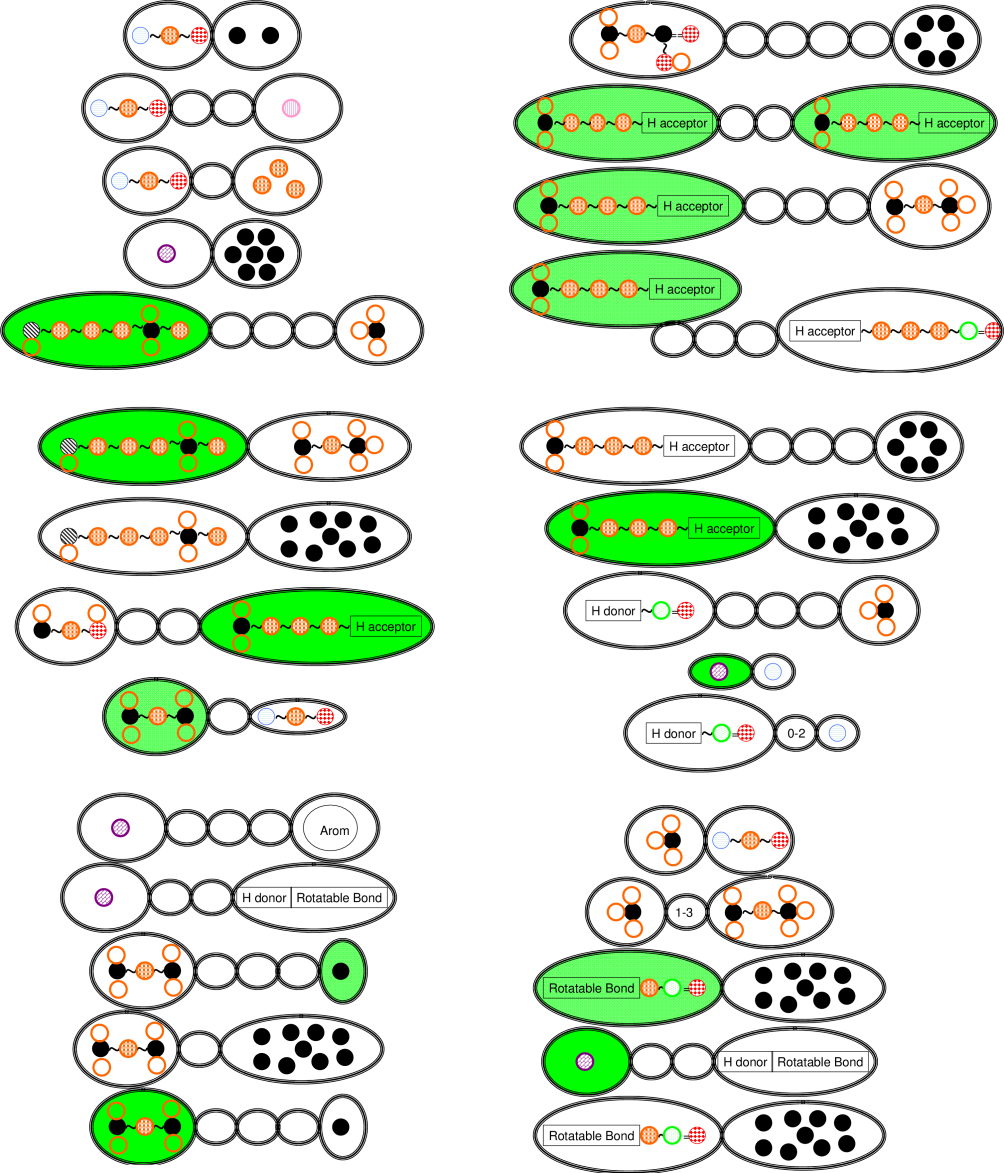
**Descriptors with high correlation to peptides' activity in SPAD**. The range of them is from 3 to 6 amino acids.

**Figure 5:**
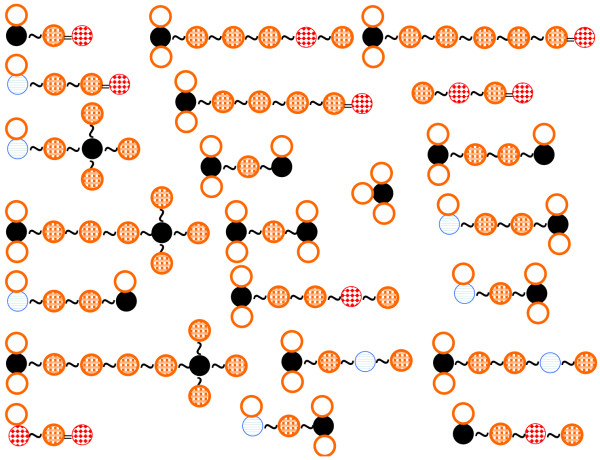
**Descriptors with high correlation to peptides' activity in APH**. The range of them is from 3 to 6 atoms. Its length is shorter than that of SPAD.

By comparing each descriptor, we know that the range of the substructures of SPAD (Figure 4) is wider than that of APH (Figure 5). The range of APH is from 3 to 7 atoms. On the other hand, the range of SPAD is from 3 to 6 amino acid residues, which usually comprises 6-12 atoms. SPAD captures a wider range of characteristics than APH. Therefore, the range of SPAD is more appropriate for capturing properties of peptides than that of APH.

#### Capturing Area of APH and SPAD in active peptides

**Figure 6:**
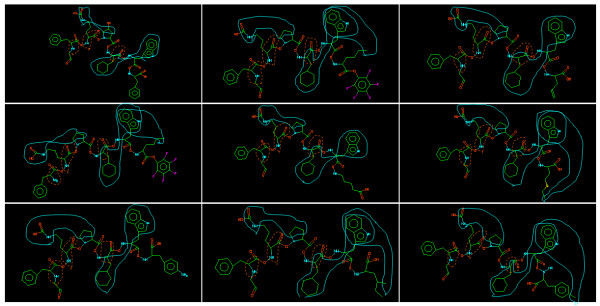
**Mapping of representative descriptors with high entropy of SPAD and APH to C5a active peptide**. Curve indicates SPAD and dotted curve indicates APH. There are few overwrapped regions between two descriptors.

In the case of SPAD (curve in Figure 6), *x *∈ *Z *or *x *∈ Z* where *x *denotes a substructure. We show substructures *x *∈ *Z *with high correlation to peptides' activity. In case of APH (dotted curve in Figure 6), we show substructures with high correlation to peptides' activity. There are few overwrapped regions between SPAD and APH. SPAD and APH capture different regions complementarily. APH inclines to capturing a component of a peptide. On the other hand, SPAD descriptor inclines to capturing a relationship of side chains between two amino acid residues.

#### Definition of dataset for similarity search with Tanimoto coefficient

Peptides are classified in three categories:

• non-active: negative peptides.

• active reference: positive peptides which are the basis of similarity search with Tanimoto coefficient.

• active: positive peptides except for active reference.

All peptides were ordered by descendent ordering with Tanimoto coefficient.

#### Comparison of the performance of SPAD with APH

When the structure of two peptides is similar and a descriptor captures a whole structure or property of peptides, these two features have similar sequences of bits. As a result, Tanimoto coefficient between these peptides becomes large. Structures of active peptides for a target protein are usually similar to each other because the pocket of target protein is same. When we describe peptides with a descriptor capturing whole peptides' structures or properties, Tanimoto coefficient between any two active peptides is larger.

Oppositely, Tanimoto coefficient between an active peptide and a non-active peptide is smaller because these two features are different to each other. However, if we describe peptides with a poor descriptor, we cannot always measure the similarity of peptides with Tanimoto coefficient. Poor descriptors break the similarity of structures at mapping to features. Therefore, Tanimoto coefficient is an indicator of the descriptor's performance.

**Figure 7:**
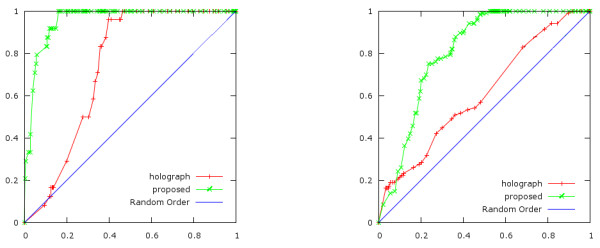
**Enrichment factor with Tanimoto coefficient**. C5a case (Left) and kinase inhibitor case (Right). The horizontal axis indicates the percentage of peptides ordered by descendent ordering with Tanimoto coefficient. The vertical axis indicates the percentage of active peptides in this ordering. The random line (diagonal line) indicates theoretically obtained curve in case of random ordering. 'x' dotted line shows the performance of SPAD and '+' dotted line shows the performance of APH. In both case, the enrichment factor of SPAD is much higher than that of APH.

All peptides are ordered by descendent ordering with Tanimoto coefficient. Then, we count the number of active peptides with this ordering. Figure 7 shows the enrichment factor with Tanimoto coefficient. The horizontal-axis and the vertical-axis is defined as follows.

• The horizontal-axis

$$\frac{The\;number\;of\;peptides\;with\;Tanimoto\;Coefficient\geq \alpha }{The\;number\;of\;all\;peptides}$$

• The vertical-axis

$$\frac{The\;number\;of\;active\;peptides\;with\;Tanimoto\;Coefficient\geq \alpha }{The\;number\;of\;active\;peptides}$$

The graph increases more rapidly as active peptides have larger Tanimoto coefficient than non-active peptides.

In both cases, C5a (left figure at Figure 7) and kinase inhibitors (right figure in Figure 7), the graph in case of SPAD is higher than the graph in case of APH. The enrichment factor with the SPAD is higher than with APH at any percentage of active peptides. Therefore, the SPAD translates similar structures to similar features more precisely than the APH. This fact means that the performance of the SPAD is higher than the performance of APH in the case of analyzing peptides' activity.

## Conclusions

It is necessary for two-dimensional QSAR of peptides that are sequences of 450 types of amino acid inductions to capture various properties with descriptors. The atom pair holographic code and substructure pair descriptor that we proposed are such descriptors. APH captures internal characters of an amino acid induction. On the other hand, SPAD captures the relationship between two amino acid inductions. SPAD captures much more information for QSAR of peptides than APH and distinguishes active peptides from non-active peptides more accurately.

## Competing interests

The authors declare that they have no competing interests.

## Authors' contributions

TO conceived the method, evaluated this method and described this manuscript. SM discussed the results and commented on the manuscript. All authors read and approved the final manuscript.
